# Effects of perioperative steroid use on surgical stress and prognosis in patients undergoing hepatectomy: a systematic review and meta-analysis of randomized controlled trials

**DOI:** 10.3389/fphar.2024.1415011

**Published:** 2024-08-15

**Authors:** Furui Zhong, Hua Yang, Xuefeng Peng, Kerui Zeng

**Affiliations:** Department of General Surgery, Zigong Fourth People’s Hospital, Zigong, Sichuan, China

**Keywords:** steroid, hepatectomy, surgical stress, prognosis, meta-analysis

## Abstract

The objective of this study was to evaluate the clinical effects of perioperative steroid hormone usage in hepatectomy patients through a comprehensive systematic review and meta-analysis. Prospective randomized controlled trials (RCTs) investigating the perioperative use of steroid hormones in hepatectomy patients were systematically searched using various databases, including PubMed, Medline, Embase, the Cochrane Library, the Chinese Biomedical Literature Database, Wanfang Data, and the CNKI database. Two researchers independently screened and extracted data from selected studies. Data analysis was performed using RevMan 5.3 software. The results revealed significantly lower levels of total bilirubin (standard mean difference [SMD] = −0.7; 95% CI: −1.23 to −0.18; and *p* = 0.009), interleukin-6 (SMD = −1.02; 95% CI: −1.27 to −0.77; and *p* < 0.001), and C-reactive protein (SMD = −0 .65; 95% CI: −1 .18 to −0.11; and *p* = 0.02) on postoperative day 1 (POD 1), as well as a reduced incidence of postoperative complications in the steroid group compared to the placebo group. No significant differences were observed between the two groups regarding alanine aminotransferase (ALT) levels, aspartic aminotransferase (AST) levels, or specific complications such as intra-abdominal infection (*p* = 0.72), wound infection (*p* = 0.1), pleural effusion (*p* = 0.43), bile leakage (*p* = 0.66), and liver failure (*p* = 0.16). The meta-analysis results indicate that perioperative steroid usage can effectively alleviate liver function impairment and inflammation response following hepatectomy while improving patient prognosis.

## Introduction

Hepatocellular carcinoma (HCC) is a prevalent malignant tumor, and surgical resection is the primary treatment option (European Association For The Study Of The Liver and European Organisation For Research And Treatment Of Cancer European Organisation For Research And Treatment Of Cancer, 2012). Hepatectomy, as a surgical procedure, imposes significant trauma. Intraoperative ischemia–reperfusion injury and stress response trigger the activation of various inflammatory pathways in the body, leading to the excessive release of inflammatory factors that subsequently cause systemic inflammatory response and tissue damage (Jiménez-Castro et al., 2019; Senoner et al., 2019). Excessive inflammatory response can further exacerbate liver injury after hepatectomy, increasing the incidence of liver dysfunction, coagulation dysfunction, perioperative complications, and mortality (Cata et al., 2017). Therefore, reducing the surgical stress response and ischemia–reperfusion injury is crucial for promoting prognosis (Cannistrà et al., 2016).

Steroid hormones can regulate gene expression to provide negative feedback for immune regulation (Scheinman et al., 1995) and inhibit lysosomal peroxidation to preserve cell membrane integrity (Serafín et al., 2004; Whitehouse, 2011), thereby stimulating the anti-inflammatory response of the body. Steroid hormones have been shown to reduce ischemia–reperfusion injury and the release of inflammatory cytokines (Hayashi et al., 2011). However, their use carries considerable risks, such as delayed wound healing, increased hyperglycemia postoperative infection risk, and reactivation of hepatitis viruses (Polderman et al., 2019). These side effects are particularly noteworthy in liver surgery; thus, it remains uncertain whether reducing the stress response translates into reduced perioperative complications. Although relevant meta-analyses have addressed this topic (Li et al., 2015; Yang et al., 2019), with new evidence being published (Onoe et al., 2021; Bressan et al., 2022), it becomes necessary to include the latest research findings for a comprehensive meta-analysis. Therefore, we conducted this meta-analysis to reassess the clinical value associated with perioperative steroid hormone usage in patients undergoing hepatectomy.

## Methods

### Search strategies

This study was conducted according to the Preferred Reporting Items for Systematic reviews and Meta-Analyses (PRISMA) guidelines (Liberati et al., 2009). Two researchers (TZ and YW) independently searched the literature on the application of steroids in hepatectomy patients through PubMed, Embase, the Cochrane Library, Medline, and Web of Science databases. The search criteria are set to a free combination of the following terms: “hepatectomy,” “liver resection,” “hepatic resection,” “steroids,” “corticosteroids,” “methylpred*,” and “hydrocortisone.” No restriction was scheduled for publication date or journal category. The literature search was limited to English and Chinese articles published before 01 November 2023. We also examined the list of references contained in the study to identify undetected relevant studies.

### Inclusion and exclusion criteria

The inclusion criteria were as follows: (1) randomized controlled trials (RCTs); (2) evaluation of the improvement of surgical stress and prognosis after the perioperative use of steroids in patients undergoing hepatectomy; and (3) outcome indicators should include those reflecting the surgical stress response (postoperative liver function and inflammatory response) and prognostic indicators of patients. The exclusion criteria were as follows: (1) study without a control group; (2) case reports, abstracts, conference reports, or animal experiments; and (3) it is not a full-text study, and the abstract does not provide sufficient information.

### Data extraction

Article selection and data extraction were performed by two researchers (TZ and YW), and if the two authors could not reach an agreement on the inclusion or exclusion of an article, the issue was resolved through consultation with the third author (HZ). After data extraction was completed, the data were reviewed by the author (HZ), and if there was any difference, the data were re-extracted and then analyzed and discussed. For studies that did not provide relevant data directly, we attempted to contact the authors of the original study but were unsuccessful. Therefore, according to the method proposed by Gheibi et al. (2019), for research that does not provide data directly but presents them in graphical form, we use Adobe Photoshop for advanced data extraction by setting the coordinate axes and then plotting points to obtain coordinates, ultimately obtaining the mean and standard deviation. The following details were extracted from the included studies: baseline data (first author, year of publication, study design, number of cases, age, and intervention mode); postoperative liver function levels and inflammatory indicators (total bilirubin [TB], alanine aminotransferase [ALT], glutamic oxaloacetic aminotransferase [AST], C-reactive protein [CRP], interleukin-6 [IL-6], and prothrombin time [PT]); and clinical outcome indicators (postoperative length of stay and complications).

### Quality assessment

Two researchers (TZ and YW) independently assessed and examined the included studies based on the Cochrane Collaboration bias risk assessment tool to ensure consistency and check for bias risk. Seven projects were considered, namely, random sequence generation, allocation concealment, blinding of participants and personnel, blinding of outcome assessment, incomplete outcome data, selective reporting, and other biases. Each project was categorized as having high, low, or unclear risk.

### Data analysis

The meta-analysis was conducted using RevMan 5.3 software provided by Cochrane. For dichotomous variables, the odds ratio (OR) and 95% CI were used as the statistical measures for effect analysis. In the case of continuous variables, the standard mean difference (SMD) and 95% CI were used as the statistics for assessing their effects. The Mantel–Haenszel test was utilized to examine heterogeneity among the included studies, with I^2^ ≤ 25% indicating low heterogeneity, 25 ≥ I^2^ ≤ 50% indicating moderate heterogeneity, and I^2^ <50% indicating high heterogeneity. A fixed-effects model was applied when there was low or moderate heterogeneity; otherwise, a random-effects model was adopted. Sensitivity analysis was performed using the one-out method to evaluate the robustness of the analysis. The publication bias in this study was assessed through a funnel plot based on primary outcomes. Statistical significance for the overall effect was considered when the *p*-value <0.05 in all analyses.

## Results

### Study identification and selection

**FIGURE 1:**
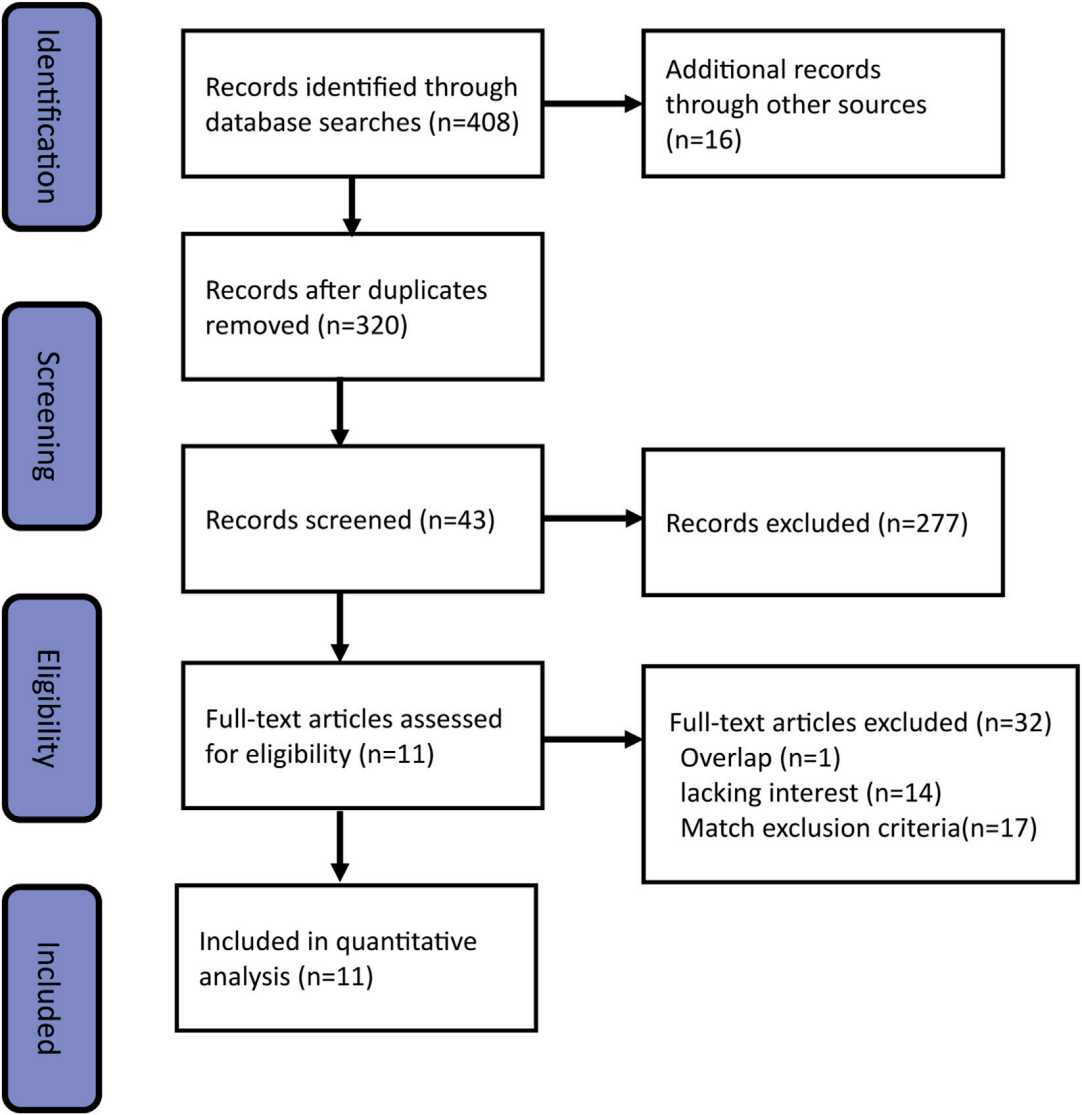
PRISMA flowchart of the literature selection.

The process of retrieving results is shown in Figure 1. According to the designed retrieval strategy, 424 relevant studies were identified after removing duplicates. By reviewing titles and abstracts, 43 potentially relevant papers were retained. Of the remaining 43 studies, 32 were excluded after full-text analysis for reasons such as center or patient cohort overlap (1 study), lack of interesting results (14 studies), or meeting one of the exclusion criteria (17 studies). Finally, a total of 11 studies (Yamashita et al., 2001; Muratore et al., 2003; Pulitanò et al., 2007; Schmidt et al., 2007; Zhai et al., 2010; Hayashi et al., 2011; Zi et al., 2015; Donadon et al., 2016; Hasegawa et al., 2020; Onoe et al., 2021; Bressan et al., 2022) were selected for the meta-analysis.

### Study characteristics

The basic and clinical characteristics of the included studies are shown in Tables 1, 2. All studies were published between 2001 and 2022, involving 895 patients undergoing hepatectomy, of which 451 patients used steroid hormones during the perioperative period, and the remaining 444 patients used placebo. Of these 11 studies, 4 were conducted in Japan, 3 in Italy, 2 in China, and 1 each from Germany and Canada. Both the steroid and placebo groups in each study were from the same single or multiple centers during the same period.

**TABLE 1:** Baseline characteristics and steroid protocol of the included studies.

Author	Year	Country	Number		Age (year)		Sex (M/F)		Steroid protocol
			Steroid	Placebo	Steroid	Placebo	Steroid	Placebo	
Yamashita	2001	Japan	17	16	60.3 ± 1.8	56.8 ± 3.9	13/4	11/5	Methylprednisolone (500 mg) intravenously, 2 h before surgery
Muratore	2003	Italy	25	28	65.4 ± 10.8	64.1 ± 11.7	17/8	11/17	Methylprednisolone (30 mg/kg) intravenously, 30 min before surgery
Schmidt	2007	Germany	10	10	57*	65*	3/7	4/6	Methylprednisolone (30 mg/kg) intravenously, 90 min before surgery
Pulitan’o	2007	Italy	36	37	61.8 ± 14.2	63 ± 13.5	22/14	23/14	Methylprednisolone (500 mg) + saline (100 mL) infusion, before anesthesia induction
Zhai	2010	China	30	30	51 ± 11.2		47/13		Hydrocortisone sodium succinate (100 mg) intravenously, continuous 5 days after operation
Hayashi	2011	Japan	102	98	69 ± 10.5	70 ± 11.7	NR	NR	Hydrocortisone (500 mg) immediately before hepatic pedicle clamping, 300 mg on POD 1, 200 mg on POD 2, and 100 mg on POD 3
Zi	2015	China	40	39	57.5 ± 8.8	57.5 ± 10.3	23/17	17/22	Methylprednisolone (500 mg) intravenously, before liver resection begins
Donadon	2016	Italy	16	16	65 ± 13.2	63 ± 12.5	10/6	9/7	Methylprednisolone (500 mg) + 5% glucose (250 mL) infusion, 1 h before surgery
Hasegawa	2020	Japan	50	50	67 ± 3.7	68 ± 3.2	30/20	31/19	Methylprednisolone (500 mg), before anesthesia induction
Onoe	2021	Japan	48	46	70 ± 11	71 ± 11.2	29/19	31/15	Hydrocortisone (500 mg) immediately before hepatic pedicle clamping, 300 mg on POD 1, 200 mg on POD 2, and 100 mg on POD 3
Bressan	2022	Canada	77	74	63.9*	62.4*	47/30	39/35	Methylprednisolone (500 mg) intravenously, during anesthesia induction

*Data are expressed as the mean.

M, male; F, female; NR, not reported; POD, postoperative day.

**TABLE 2:** Clinical characteristics of the included studies.

First author	Major resection		Resection technique	Vascular control	Operative time		Ischemic time		Blood loss	
	Steroids	Placebo			Steroid	Placebo	Steroid	Placebo	Steroid	Placebo
Yamashita	5	6	NR	PTC or HVE or TVE	338 ± 21	352 ± 14	NR	NR	892 ± 106	822 ± 55
Muratore	13	15	KC or UD	PTC	NR	NR	41.4 ± 15.9	37.3 ± 17.8	322 ± 261	294 ± 271
Schmidt	6	5	UD	None used	222*	252*	NR	NR	340*	780*
Pulitan’o	24	24	UD and US	PTC	408 ± 55	440 ± 65	52.4 ± 17.2	43 ± 14.5	621 ± 125	621 ± 92.5
Zhai	8		NR	PTC	NR	NR	16 ± 5.2		399 ± 612	
Hayashi	11	15	NR	PTC	330 ± 167	316 ± 140	72 ± 61	60 ± 50	324 ± 393	257 ± 490
Zi	22	12	NR	PTC	342.3 ± 129.7	353.2 ± 168.3	NR	NR	481 ± 415	496 ± 391
Donadon	7	5	CM and LM	PTC	383 ± 77	351 ± 103	83 ± 29	33 ± 35	275 ± 225	200 ± 175
Hasegawa	10	11	CM and MC	PTC	215 ± 31	223 ± 28	65 ± 7.5	60 ± 9.7	52 ± 30	30 ± 15
Onoe	32	31	US	PTC	517 ± 158	515 ± 151	63 ± 29.7	63 ± 45	1,010 ± 591	907 ± 1,163
Bressan	47	37	NR	PTC	134 ± 32	148 ± 28	NR	NR	339 ± 242	395 ± 266

*Data are expressed as the mean.

NR, not reported; PTC, portal triad clamping; UD, ultrasonic dissector; KC, Kelly-clysis; US, ultrasonic shears; HVE, hemi-hepatic vascular exclusion; TVE, total vascular exclusion.

### Assessment of research quality

The Cochrane Collaboration network bias risk tool was used to evaluate the quality of the included studies. Six studies (Yamashita et al., 2001; Pulitanò et al., 2007; Schmidt et al., 2007; Zhai et al., 2010; Hasegawa et al., 2020; Bressan et al., 2022) reported randomization sequences generated by computer programs, and the remaining studies were unclear. Eight studies (Yamashita et al., 2001; Muratore et al., 2003; Pulitanò et al., 2007; Schmidt et al., 2007; Zhai et al., 2010; Hayashi et al., 2011; Hasegawa et al., 2020; Onoe et al., 2021) reported allocation concealment schemes, including the envelope method and central randomization. Seven studies (Muratore et al., 2003; Pulitanò et al., 2007; Zhai et al., 2010; Zi et al., 2015; Donadon et al., 2016; Onoe et al., 2021; Bressan et al., 2022) described the blinding of subjects and personnel, and all the studies were considered had blinding of outcome assessors due to objective outcomes. Selective outcome reporting and other sources of bias were not identified. The quality evaluation of the included studies is shown in Supplementary Figure S1.

### Effects of steroids on surgical stress

**FIGURE 2:**
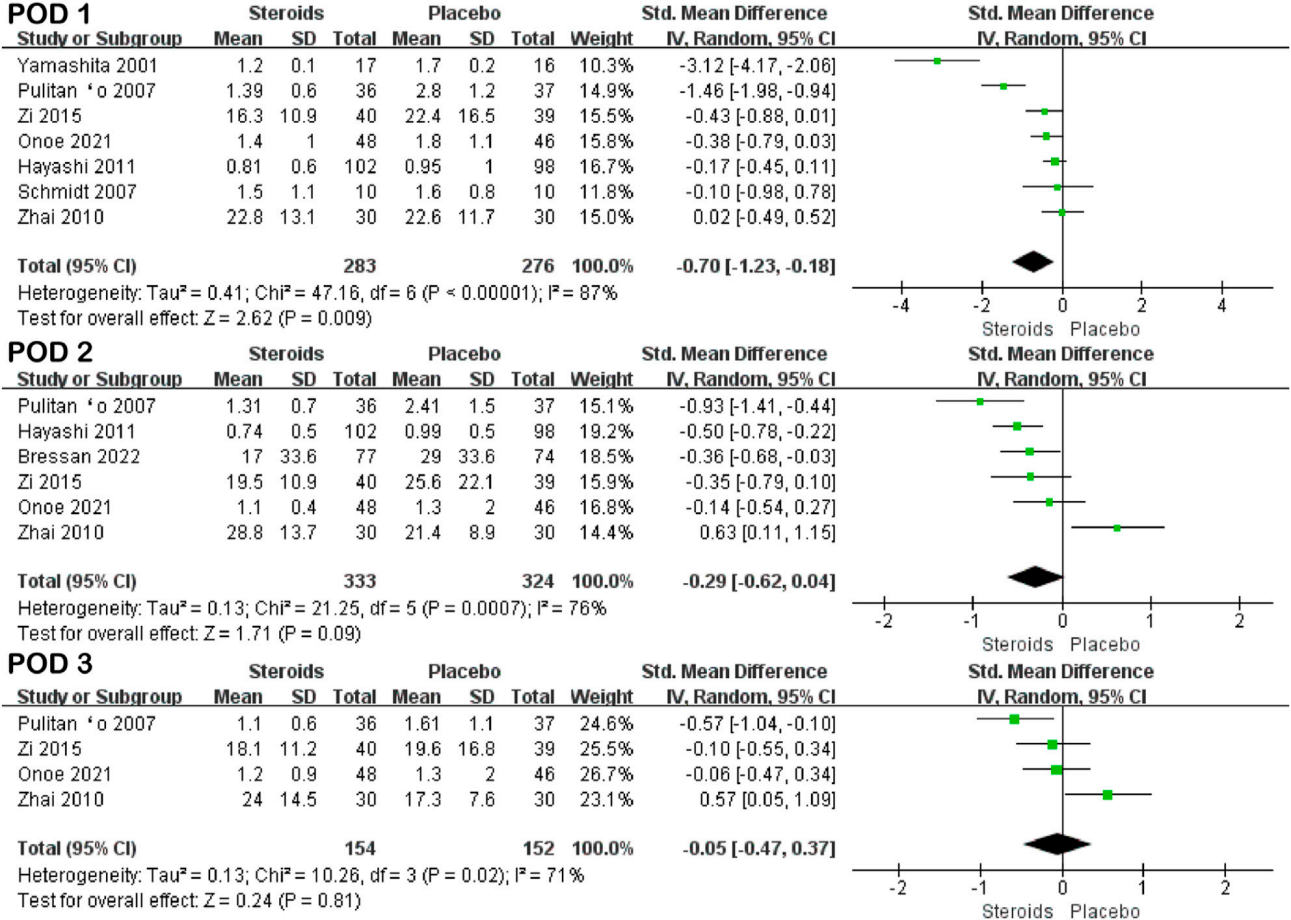
Forest plot of the pooled data for total bilirubin levels on PODs 1, 3, and 5.

Seven studies (Yamashita et al., 2001; Pulitanò et al., 2007; Schmidt et al., 2007; Zhai et al., 2010; Hayashi et al., 2011; Zi et al., 2015; Onoe et al., 2021), six studies (Pulitanò et al., 2007; Zhai et al., 2010; Hayashi et al., 2011; Zi et al., 2015; Onoe et al., 2021; Bressan et al., 2022), and four studies (Pulitanò et al., 2007; Zhai et al., 2010; Zi et al., 2015; Onoe et al., 2021) reported total bilirubin on PODs 1, 3, and 5, respectively. Due to high heterogeneity (I^2^ = 87%, I^2^ = 76%, and I^2^ = 71%), the random-effects model was adopted for the pooled analysis. The meta-analysis showed that in patients undergoing hepatectomy, the total bilirubin level on POD 1 was significantly lower in the steroid group than in the control group (SMD = −0. 7; 95% CI: −1.23 to −0.18; and *p* = 0.009) (Figure 2; Table 3). However, there was no significant difference in total bilirubin levels between the two groups on PODs 3 and 5 (SMD = −0. 29; 95% CI: −0.62 to −0.04; and *p* = 0.09 and SMD = −0. 05; 95% CI: −0.47 to 0.37; and *p* = 0.81) (Figure 2; Table 3).

**TABLE 3:** Forest plot statistics summary by outcome.

Outcome		Number	Heterogeneity (I^2^) (%)	SMD, 95% CI	Z-value	*p*-value
TB	POD 1	7	87	−0.7 (−1.23, −0.18)	2.62	0.009
	POD 3	6	76	−0.29 (−0.62, −0.04)	4.24	<0.001
	POD 5	4	71	−0.05 (−0.47, 0.37)	0.24	0.81
ALT	POD 1	7	88	−0.1 (−0.27, 0.07)	1.15	0.25
	POD 3	4	36	−0.09 (−0.37, 0.19)	0.61	0.54
	POD 5	3	0	−0.04 (−0.29, 0.22)	0.27	0.79
AST	POD 1	7	82	−0.03 (−0.46, 0.4)	0.12	0.9
	POD 3	4	41	−0.09 (−0.99, 0.2)	0.6	0.55
	POD 5	3	0	0.11 (−0.14, 0.37)	0.86	0.39
CRP	POD 1	4	70	−0.65 (−1.18, −0.11)	2.37	0.02
	POD 2	6	89	−1.31 (−1.94, −0.68)	4.06	<0.001
IL-6	POD 1	5	22	−1.02 (−1.27, −0.77)	7.95	<0.001
	POD 3	3	0	−0.99 (−1.36, −0.61)	5.19	<0.001
PT	POD 1	3	91	−0.34 (−1.43, 0.74)	0.62	0.54
	POD 2	4	0	−0.29 (−0.48, −0.09)	2.90	0.004

Seven studies (Yamashita et al., 2001; Pulitanò et al., 2007; Schmidt et al., 2007; Zhai et al., 2010; Hayashi et al., 2011; Zi et al., 2015; Onoe et al., 2021), four studies (Pulitanò et al., 2007; Zhai et al., 2010; Zi et al., 2015; Onoe et al., 2021), and three studies (Zhai et al., 2010; Zi et al., 2015; Onoe et al., 2021) described ALT and AST levels on PODs 1, 3, and 5, respectively. The meta-analysis showed that there was no significant difference in ALT and AST levels between the steroid and placebo group after hepatectomy (*p* > 0.05), and the relevant results are shown in Table 3.

**FIGURE 3:**
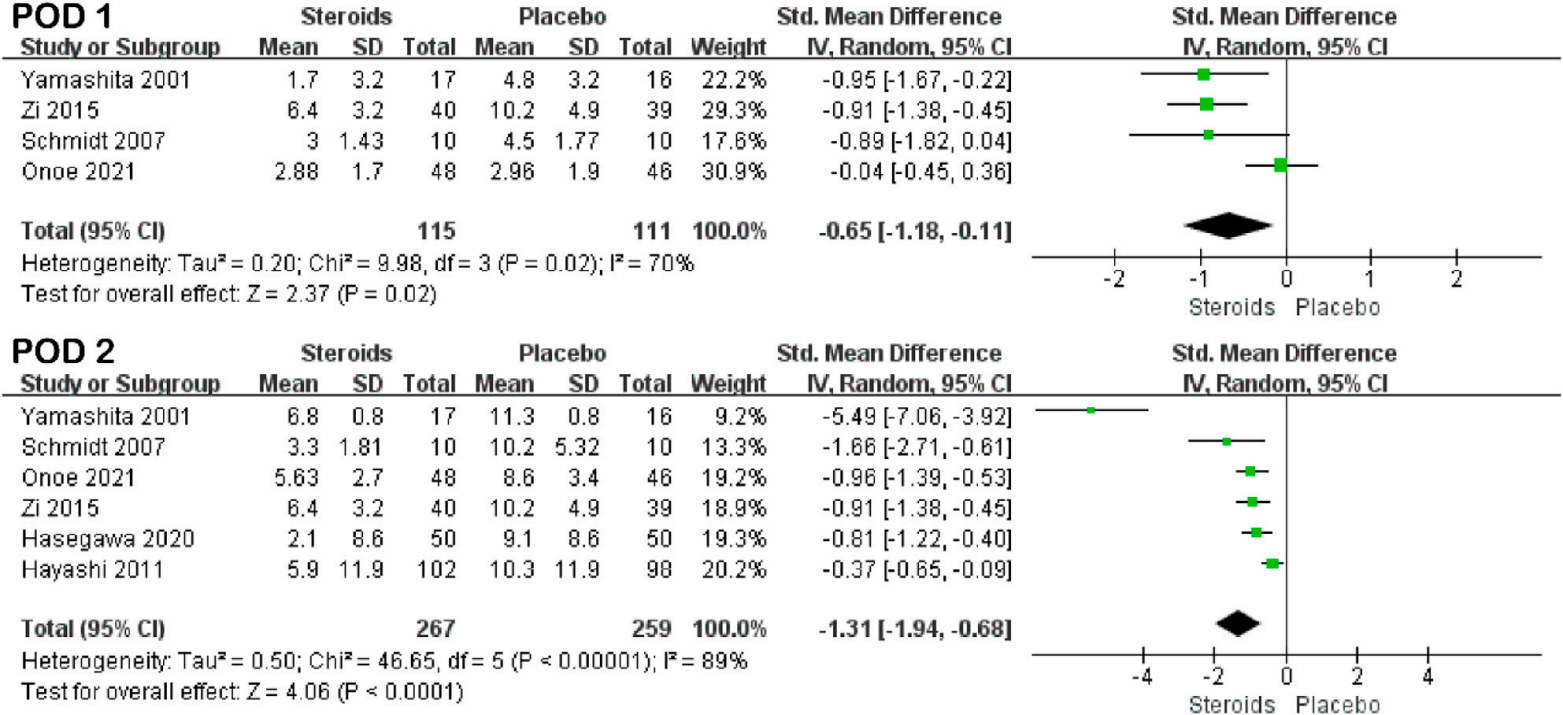
Forest plot of the pooled data for CRP levels on PODs 1 and 2.

Serum C-reactive protein levels on PODs 1 and 2 were reported in four studies (Yamashita et al., 2001; Schmidt et al., 2007; Zi et al., 2015; Onoe et al., 2021) and six studies (Yamashita et al., 2001; Schmidt et al., 2007; Hayashi et al., 2011; Zi et al., 2015; Hasegawa et al., 2020; Onoe et al., 2021). Because of the high heterogeneity (I^2^ = 70% and I^2^ = 89%), the random-effects model was used for the pooled analysis. The results of the meta-analysis showed that the C-reactive protein level in the steroid group was significantly lower than that in the control group on PODs 1 and 2 (SMD = −0.65; 95% CI: −1.18 to −0.11; and *p* = 0.02 and SMD = −1.31; 95% CI: −1.94 to −0.68; and *p* < 0.001) (Figure 3; Table 3).

**FIGURE 4:**
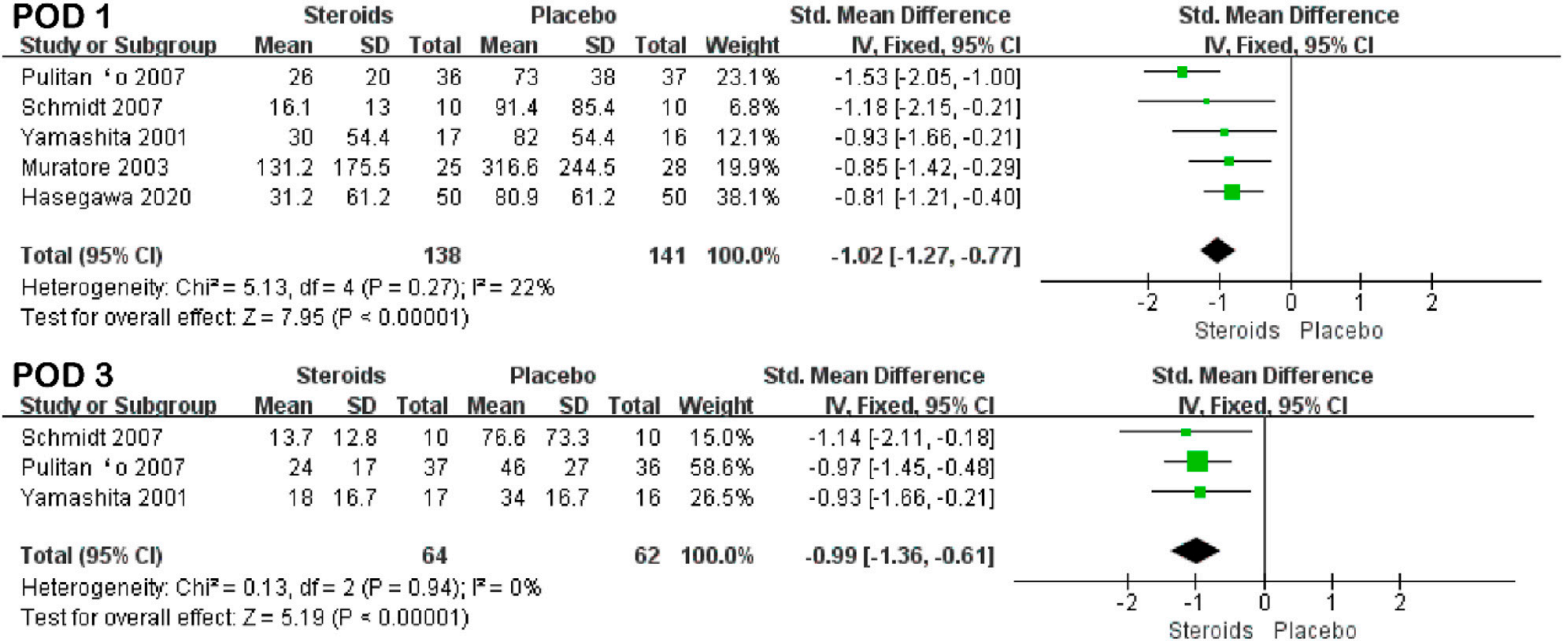
Forest plot of the pooled data for IL-6 levels on PODs 1 and 3.

The interleukin-6 index was reported in five studies (Yamashita et al., 2001; Muratore et al., 2003; Pulitanò et al., 2007; Schmidt et al., 2007; Hasegawa et al., 2020) and three studies (Yamashita et al., 2001; Pulitanò et al., 2007; Schmidt et al., 2007) on PODs 1 and 3, respectively. Due to the low heterogeneity (I^2^ = 22% and I^2^ = 0%), the fixed-effects model was used for the pooled analysis. The results of the meta-analysis indicated that the IL-6 level in the steroid group was significantly lower than that in the placebo group on PODs 1 and 3 (SMD = −1.02; 95% CI: −1.27 to −0.77; and *p* < 0.001 and SMD = −0.99; 95% CI: −1.36 to −0.61; and *p* < 0.001) (Figure 4; Table 3).

**FIGURE 5:**
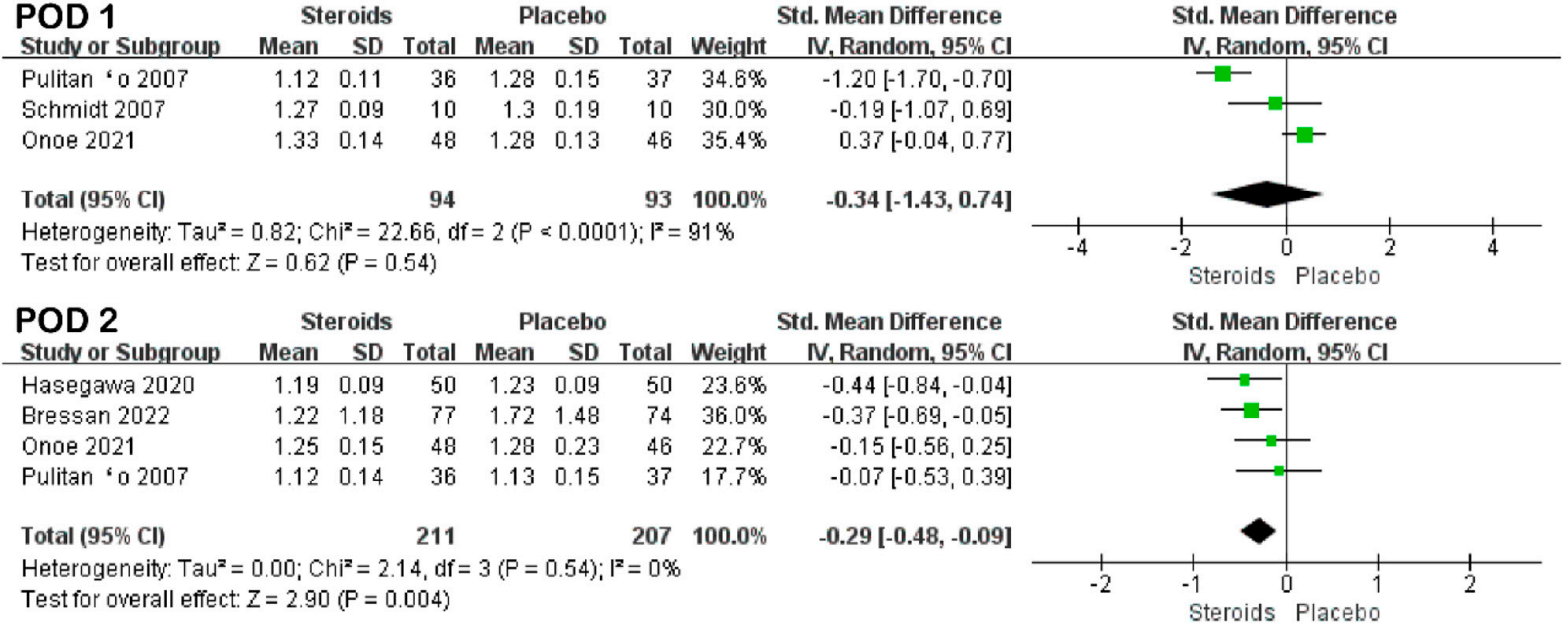
Forest plot of the pooled data for PT on PODs 1 and 2.

**FIGURE 6:**
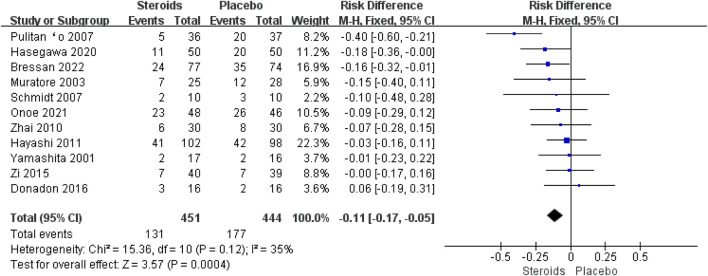
Forest plot of the pooled data for postoperative hospital stay.

Prothrombin time was mentioned in three studies (Pulitanò et al., 2007; Schmidt et al., 2007; Onoe et al., 2021) and four studies (Pulitanò et al., 2007; Hasegawa et al., 2020; Onoe et al., 2021; Bressan et al., 2022) on PODs 1 and 2, respectively. The results of the meta-analysis showed that there was no significant difference in prothrombin time between the steroid and placebo groups on POD 1 (SMD = −0.34; 95% CI: −1.43 to 0.74; and *p* = 0.54) (Figure 6; Table 3), but the prothrombin time of the steroid group was significantly lower than that of the placebo group on POD 2 (SMD = −0.29; 95% CI: −0.48 to −0.09; and *p* = 0.004) (Figure 5; Table 3).

### Effect of steroids on clinical prognosis

All 11 studies (Yamashita et al., 2001; Muratore et al., 2003; Pulitanò et al., 2007; Schmidt et al., 2007; Zhai et al., 2010; Hayashi et al., 2011; Zi et al., 2015; Donadon et al., 2016; Hasegawa et al., 2020; Onoe et al., 2021; Bressan et al., 2022) reported postoperative hospital stays. Due to the high heterogeneity (I^2^ = 65%), the random-effects model was used for the combined analysis. The results of the meta-analysis showed that there was no significant difference in postoperative hospital stay between the two groups (SMD = −0.1; 95% CI: −0.34 to 0.14; and *p* = 0.41) (Figure 6).

**FIGURE 7:**
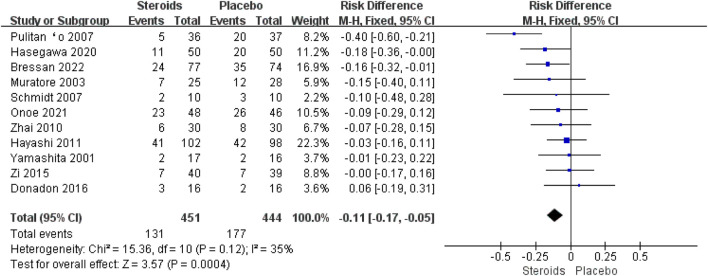
Forest plot of the pooled data for overall postoperative complications.

All 11 studies (Yamashita et al., 2001; Muratore et al., 2003; Pulitanò et al., 2007; Schmidt et al., 2007; Zhai et al., 2010; Hayashi et al., 2011; Zi et al., 2015; Donadon et al., 2016; Hasegawa et al., 2020; Onoe et al., 2021; Bressan et al., 2022) described the overall postoperative complications. Due to the high heterogeneity (I^2^ = 35%), the fixed-effects model was used for the pooled analysis. The meta-analysis showed that the overall complications after hepatectomy in the steroid group were significantly lower than those in the placebo group (OR = −0.11; 95% CI: −0.17 to −0.05; and *p* < 0.001) (Figure 7).

**FIGURE 8:**
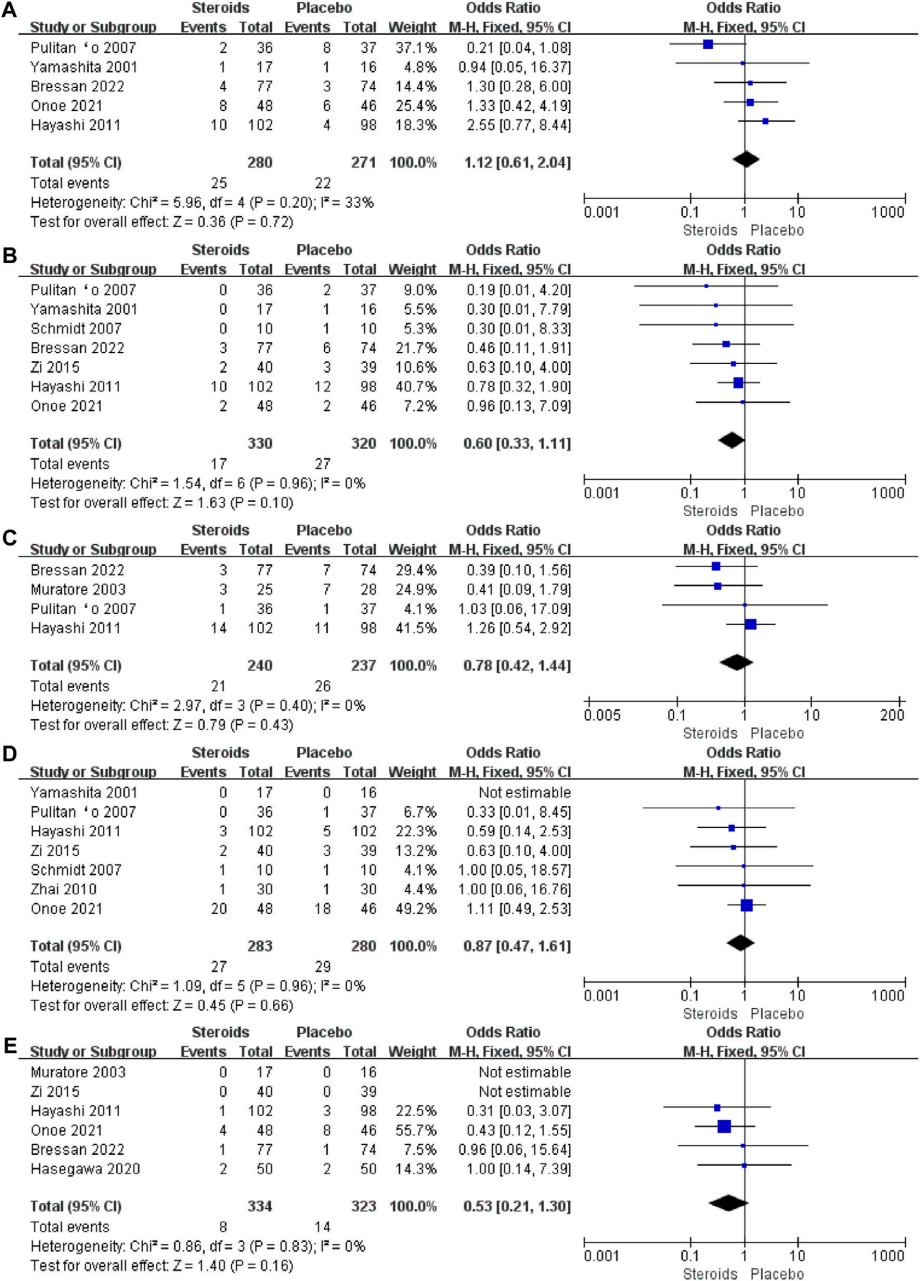
Forest plot of the pooled data for each specific complication: **(A)** intra-abdominal infection; **(B)** wound infection; **(C)** pleural effusion; **(D)** bile leakage; and **(E)** liver failure.

To determine the postoperative complications that benefit from the use of steroids in patients undergoing hepatectomy, we further analyzed the incidence of each specific complication. The results of the meta-analysis showed that there was no significant difference in the incidence of postoperative intra-abdominal infection (OR = 1.12; 95% CI: 0.61 to 2.04; and *p* = 00.72) (Figure 8A), wound infection (OR = 0.6; 95% CI: 0.33 to 1.11; and *p* = 0.1) (Figure 8B), pleural effusion (OR = 0.78; 95% CI: 0.42 to 1.44; and *p* = 0.43) (Figure 8C), bile leakage (OR = 0.87; 95% CI: 0.47 to 1.61; and *p* = 0.66) (Figure 8D), and liver failure (OR = 0.53; 95% CI: 0.21 to 1.3; and *p* = 0.16) (Figure 8E) between the steroid and placebo groups. The results are given in Table 3. Among all the included studies, only the study by Bressan et al. (2022) reported postoperative deaths, with four patients dying within 90 days after surgery, while no postoperative death was reported in the other studies.

### Sensitivity analysis and publication bias

The sensitivity analysis was performed by eliminating one study in each turn; the results of total bilirubin, AST, and ALT levels, hospital stay, and complications remained consistent with the original outcomes. The sensitivity analysis for postoperative complications was attributed to the study by Yamashita et al. (2001). After excluding this study, the results of the combined analysis changed significantly (SMD = −0.65; 95% CI: −1.18 to −0.11; and *p* = 0.09). Supplementary Figure S2 shows the funnel plot of the overall postoperative complications, from which one study did not fall within the 95% confidence interval range, and therefore, this result may have publication bias.

## Discussion

Hepatectomy is considered one of the more intricate procedures in the field of surgery. With a comprehensive understanding of liver anatomy, intraoperative bleeding-related fatalities have become rare, while postoperative stress reaction-induced organ dysfunction has emerged as the primary factor influencing patient prognosis (Nguyen et al., 2009). The liver serves as the main producer of inflammatory mediators during traumatic stress (inflammatory mediators of liver ischemia). Compared to other surgical operations, hepatectomy involves complex surgical techniques, prolonged operation duration, and significant intraoperative blood loss, resulting in substantial damage to the liver (S and J, 2011). Intraoperative blood flow occlusion can easily lead to ischemia–reperfusion injury in the liver (Jiménez-Castro et al., 2019). Simultaneously, the postoperative stress response triggers an abundance of inflammatory mediators that disrupt the internal environment of the body and cause acute liver reactions (Søreide and Deshpande, 2021). Therefore, controlling excessive inflammatory responses caused by surgical stress is crucial for enhancing hepatectomy safety and improving patient prognosis (Boermeester et al., 1995).

Steroid hormones exert potent anti-inflammatory effects through various pathways (Scheinman et al., 1995). Srinivasa et al. (2011) investigated the clinical safety and efficacy of preoperative glucocorticoid use on short-term outcomes following major abdominal surgery. Their findings demonstrated that preoperative glucocorticoid administration could reduce postoperative complications and hospital stays after major abdominal surgery. This reduction may be attributed to decreased postoperative inflammatory responses. A meta-analysis previously conducted by Yang et al. (2019) and Hai et al. (2021) also suggested that administering steroids before hepatectomy could facilitate liver function recovery and inhibit inflammation.

Our study is a continuation and update of the work by Yang et al. (2019) and Hai et al. (2021), incorporating the latest research evidence (Onoe et al., 2021; Bressan et al., 2022) and Chinese studies (Zhai et al., 2010; Zi et al., 2015). The results of our pooled analysis demonstrate significant improvements in postoperative inflammatory markers such as IL-6, CRP, and prothrombin time in patients undergoing hepatectomy after steroid administration. Additionally, there was a significant reduction in the total bilirubin levels (SMD = −0.7; 95% CI: −1.23 to −0.18; and *p* = 0.009) on postoperative day 1. These findings reaffirm the potent anti-inflammatory effects of steroid hormones and their potential to enhance liver function following hepatectomy. Pro-inflammatory cytokines like IL-6 and CRP are upregulated after hepatectomy (Andus et al., 1991), leading not only to tissue damage but also an amplified inflammatory response (Boermeester et al., 1995), resulting in the cascade release of numerous inflammatory mediators and cytokines (Belkaid and Hand, 2014), posing a serious threat to patient life and health. Modulating pro-inflammatory cytokine expression can mitigate tissue damage while potentially improving patient outcomes (Tuomisto et al., 2019).

Previous concerns regarding the use of steroid hormones during hepatectomy have limited their application due to several reasons. First, their robust immunosuppressive effect may increase the risk of postoperative infections (Corcoran et al., 2021), as well as reactivate hepatitis viruses such as hepatitis B or C virus, causing liver tissue damage (Oketani et al., 2012). Second, steroid hormones may induce hypertension (Dodic et al., 1999) and hyperglycemia (Sunena and Mishra, 2022), along with peptic ulcers and various metabolic disorders (Conn and Poynard, 1994), thereby complicating diseases further. Moreover, long-term usage has been associated with water–sodium retention effects that can impose additional cardiac load, burdening the liver (Oray et al., 2016). However, as more clinical studies are published, these doubts gradually diminish, and it is increasingly recognized that the perioperative administration of steroid hormones can enhance postoperative outcomes following hepatectomy (Schmidt et al., 2007). The potential risks associated with short-term and moderate steroid use, such as immunosuppression, water–sodium retention, hypertension, and peptic ulcers, should not be overestimated (Steinthorsdottir et al., 2021). Our meta-analysis also demonstrated that the utilization of steroid hormones during hepatectomy not only improved liver function and reduced inflammation but also significantly decreased overall postoperative complications (OR = −0.11; 95% CI: −0.17 to −0.05; and *p* < 0.001).

Some limitations to this study need to be addressed. First, there was considerable heterogeneity observed in certain results. Sensitivity analysis and subgroup analysis could help identify the sources of high heterogeneity. However, due to difficulties in accessing original data, we were unable to perform subgroup analysis based on the cirrhosis background or extent of hepatectomy. Second, since some results were presented graphically in the original text without the author’s contact information available for clarification purposes, relevant data had to be extracted from figures, which may introduce measurement bias using this method alone. Third, a funnel plot analysis revealed potential publication bias regarding total postoperative complication outcome measures. Fourth, in our meta-analysis examining specific complications, it remains inconclusive which postoperative complications can be effectively mitigated by steroid hormone usage. This aspect warrants further investigation in future studies.

In conclusion, the findings of this systematic review and meta-analysis demonstrate that the administration of steroid hormones in patients undergoing hepatectomy effectively reduces postoperative liver function damage and inflammatory response while improving clinical prognosis.

## Data Availability

The original contributions presented in the study are included in the article/Supplementary Material; further inquiries can be directed to the corresponding author.
