# Supplementary material for: Effects of perioperative steroid use on surgical stress and prognosis in patients undergoing hepatectomy: a systematic review and meta-analysis of randomized controlled trials
Source: Front Pharmacol. 2024 Aug 15;15:1415011. doi: 10.3389/fphar.2024.1415011 (PMC11393686; doi:10.3389/fphar.2024.1415011)

Supplementary Figure 1. Assessment of risk of bias: (A) Risk of bias graph; (B) Risk of bias summary.


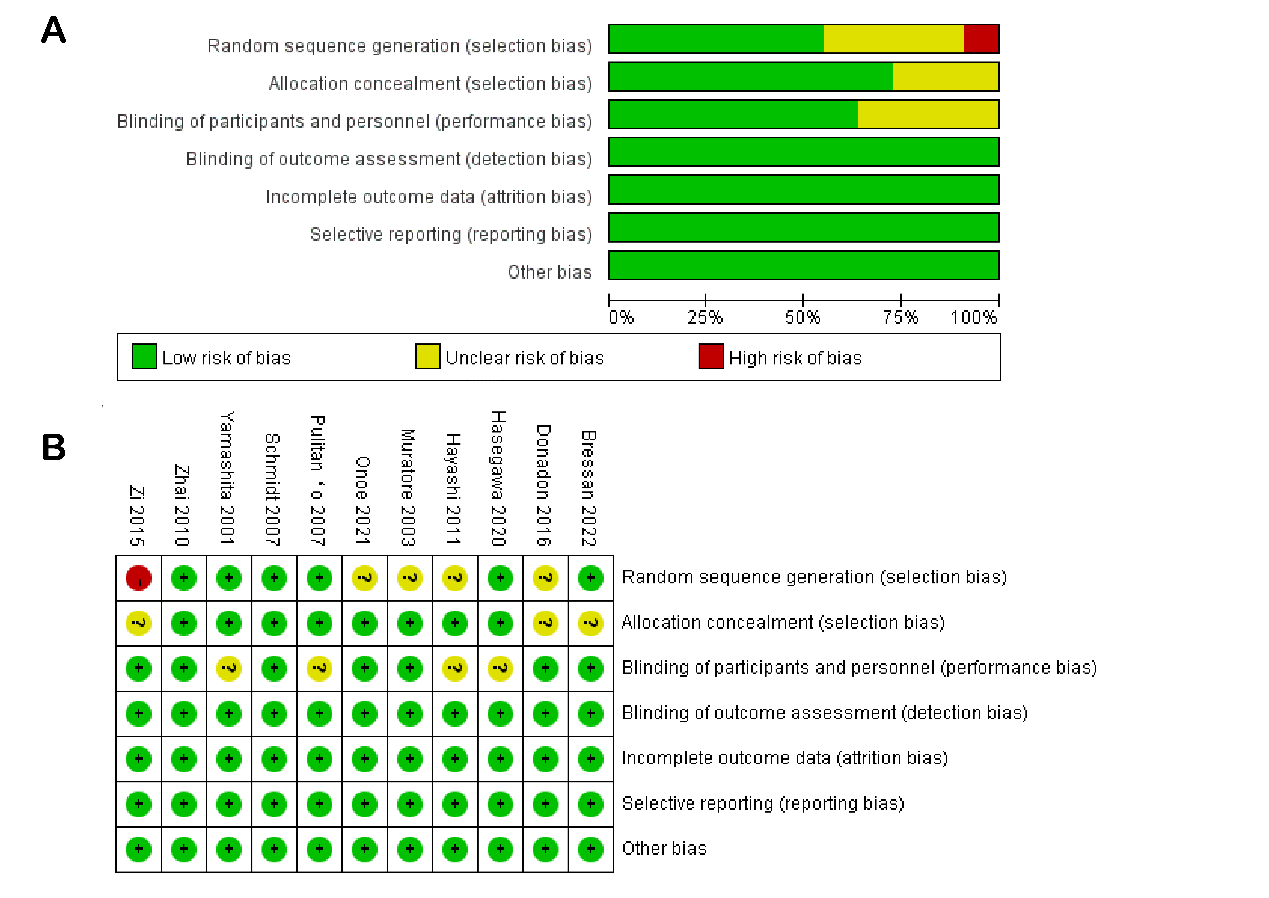


Supplementary Figure 2. Funnel plot for the publication bias.


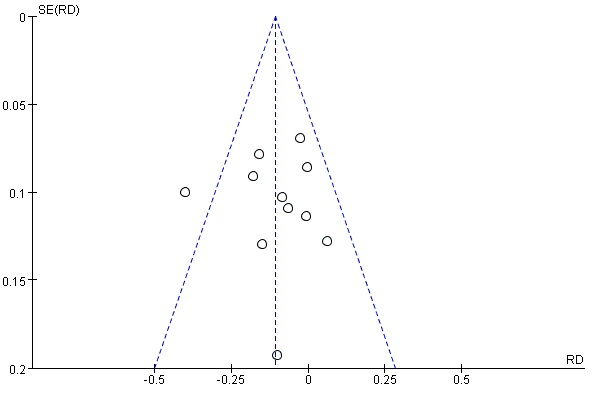

Supplement: Supplementary file 1 [file DataSheet1.docx]
